# Detecting causality from online psychiatric texts using inter-sentential language patterns

**DOI:** 10.1186/1472-6947-12-72

**Published:** 2012-07-18

**Authors:** Jheng-Long Wu, Liang-Chih Yu, Pei-Chann Chang

**Affiliations:** 1 College of Informatics, Department of Information Management, Yuan Ze University, Chung-Li, Taiwan, Republic of China

**Keywords:** Causality detection, Inter-sentential language patterns, Biomedical text mining, Natural language processing

## Abstract

**Background:**

Online psychiatric texts are natural language texts expressing depressive problems, published by Internet users via community-based web services such as web forums, message boards and blogs. Understanding the cause-effect relations embedded in these psychiatric texts can provide insight into the authors’ problems, thus increasing the effectiveness of online psychiatric services.

**Methods:**

Previous studies have proposed the use of word pairs extracted from a set of sentence pairs to identify cause-effect relations between sentences. A word pair is made up of two words, with one coming from the cause text span and the other from the effect text span. Analysis of the relationship between these words can be used to capture individual word associations between cause and effect sentences. For instance, (broke up, life) and (boyfriend, meaningless) are two word pairs extracted from the sentence pair: “I broke up with my boyfriend. Life is now meaningless to me”. The major limitation of word pairs is that individual words in sentences usually cannot reflect the exact meaning of the cause and effect events, and thus may produce semantically incomplete word pairs, as the previous examples show. Therefore, this study proposes the use of inter-sentential language patterns such as ≪broke up, boyfriend>, <life, meaningless≫ to detect causality between sentences. The inter-sentential language patterns can capture associations among multiple words within and between sentences, thus can provide more precise information than word pairs. To acquire inter-sentential language patterns, we develop a text mining framework by extending the classical association rule mining algorithm such that it can discover frequently co-occurring patterns across the sentence boundary.

**Results:**

Performance was evaluated on a corpus of texts collected from PsychPark (http://www.psychpark.org), a virtual psychiatric clinic maintained by a group of volunteer professionals from the Taiwan Association of Mental Health Informatics. Experimental results show that the use of inter-sentential language patterns outperformed the use of word pairs proposed in previous studies.

**Conclusions:**

This study demonstrates the acquisition of inter-sentential language patterns for causality detection from online psychiatric texts. Such semantically more complete and precise features can improve causality detection performance.

## Background

Online community-based services such as web forums, message boards, and blogs provide an efficient and effective way for sharing information and gathering knowledge [1-3]. In the field of mental health care, these services allow individuals to describe their life stresses and depressive problems to other Internet users or health professionals who can then make recommendations to help the subject developing the knowledge needed to seek appropriate care. Examples of these websites include Depression Forums^a^, PsychPark^b^, SA-UK^c^, WebMD^d^, and Yahoo!Answers^e^. This paper refers to this type of online post as online psychiatric texts, and their major characteristic is that they are in the form of natural language texts, featuring many cause-effect relations between sentences. Some examples of causality sentences are presented below:

(E1) *I couldn’t sleep for several days because my boss cut my salary.*

(E2) *I failed again. I felt very upset.*

(E3) *I broke up with my boyfriend. Life now is meaningless to me.*

These examples indicate three depressive problems caused by negative life events experienced by the speaker. Awareness of such cause-effect relations between sentences can improve our understanding of users’ problems and make online psychiatric services more effective. For instance, systems capable of identifying causality from online forum posts could assist health professionals in capturing users’ background information more quickly, thus decreasing response time. Additionally, a dialog system could generate supportive responses if it could understand depressive problems and their associated reasons embedded in users’ input. Recent studies also show that causality is an important concept in biomedical informatics [4], and identifying cause-effect relations as well as other semantic relations could improve the effectiveness of many applications such as question answering [5-7], biomedical text mining [8-10], future event prediction [11], information retrieval [12], and e-learning [13]. Therefore, this paper proposes a text mining framework to detect cause-effect relations between sentences from online psychiatric texts.

Causality (or a cause-effect relation) is a relation between two events: cause and effect. In natural language texts, cause-effect relations can generally be categorized as explicit and implicit depending on whether or not a discourse connective (e.g., “because”, “therefore”) is found between the cause and effect text spans [14-16]. For instance, the example sentence E1 contains an explicit cause-effect relation due to the presence of the discourse connective “because” which signals the relation. Conversely, both E2 and E3 lack a discourse connective and thus the cause-effect relation between the sentences is implicit. Traditional approaches to identifying explicit cause-effect relations have focused on mining useful discourse connectives that can trigger the cause-effect relation. Wu et al. [17] manually collected a set of discourse connectives to identify cause-effect relations from psychiatric consultation records. Ramesh and Yu [18] proposed the use of a supervised machine learning method called conditional random fields (CRFs) to automatically identify discourse connectives in biomedical texts. Inui et al. [19] used a discourse connective “*tame*” to acquire causal knowledge from Japanese newspaper articles. Although discourse connectives are useful features for identifying causality, the difficulty inherent in collecting a complete set of discourse connectives may result in this approach failing to identify the cause-effect relations triggered by unknown discourse connectives. In addition, it may also fail to identify implicit cause-effect relations that lack an explicit discourse connective between the sentences. Accordingly, other useful features and algorithms have been investigated to identify implicit causality within [20,21] and between sentences [22,23]. Efforts to identify causality within sentences have investigated features that consider sentence structure. Rink et al. [20] proposed the use of textual graph patterns obtained from parse trees to determine whether two events from the same sentence have a causal relation. Mulkar-Mehta et al. [21] introduced a theory of granularity to identify sentences containing causal relations. Features across the sentence boundary could be useful in identifying causality between sentences because such features can capture feature relationships between sentences. For instance, word pairs in which one word comes from the cause text span and the other comes from the effect text span have been demonstrated to be useful features for discovering implicit causality between sentences [22,23] because they can capture individual word associations between cause and effect sentences. In the E2 sample sentence pair, the word pair (fail, upset) helps identify the implicit cause-effect relation that holds between the two sentences.

However, within the sentences, individual words usually cannot reflect the exact meaning of the cause and effect events which, taking E3 as an example, may produce semantically incomplete word pairs such as (broke up, life), (broke up, meaningless), (boyfriend, life), and (boyfriend, meaningless). In fact, many cause and effect events can be characterized by language patterns, i.e., meaningful combinations of words. For instance, in E3, the first sentence (cause) can be characterized by a language pattern < broke up, boyfriend>, and the second sentence (effect) can be characterized by < life, meaningless>. Combining these two intra-sentential language patterns constitutes a more semantically complete inter-sentential language pattern < <broke up, boyfriend>, <life, meaningless>>. Such inter-sentential language patterns can provide more precise information to improve the performance of causality detection because they can capture the associations of multiple words within and between sentences. Therefore, this study develops a text mining framework by extending the classical association rule mining algorithm [24-28] such that it can mine inter-sentential language patterns by associating frequently co-occurred patterns across the sentence boundary. The discovered patterns are then incorporated into a probabilistic model to detect causality between sentences.

The rest of this paper is organized as follows. We first describe the framework for inter-sentential language pattern mining and causality detection. We then summarize the experimental results of and present conclusions.

## Methods

**Figure 1:**
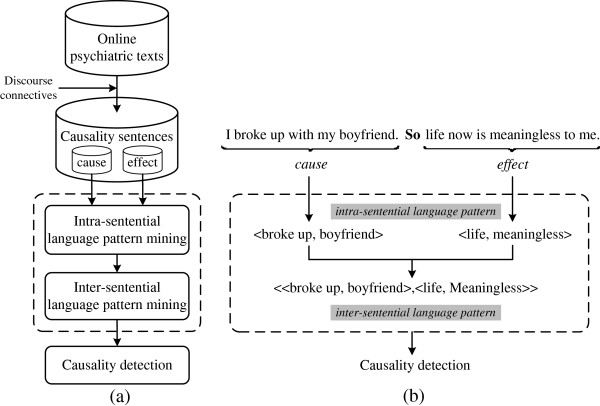
**(a) Framework of inter-sentential language patterns mining and causality detection.** (**b**) Example of inter-sentential language patterns mining.

(Figure 1(a)) illustrates the framework of inter-sentential language pattern mining and causality detection. The online psychiatric texts are a collection of forum posts collected from PsychPark (http://www.psychpark.org), a virtual psychiatric clinic maintained by a group of volunteer professionals belonging to the Taiwan Association of Mental Health Informatics [29,30]. A set of discourse connectives based on the results of previous studies [16,17] was created to select causality sentences from the online psychiatric texts. These causality sentences are then split into cause and effect text spans by removing the discourse connectives between them. For instance, in (Figure 1(b)), the sample causality sentences can be split by removing the discourse connective “so”. Next, the sets of cause and effect text spans are processed by the algorithm in two steps: intra-sentential and inter-sentential language pattern mining. Intra-sentential language pattern mining is used to discover language patterns of frequently co-occurring words within the cause and effect text spans. Once the intra-sentential language patterns are discovered, the frequently co-occurred patterns between the cause and effect text spans are then combined to form a set of inter-sentential language patterns. As indicated in (Figure 1(b)), two intra-sentential language patterns < broke up, boyfriend > and < life, meaningless > are discovered from their respective cause and effect text spans, and they constitute an inter-sentential language pattern <<broke up, boyfriend>, <life, meaningless>>. Finally, the acquired inter-sentential language patterns are used as features to detect causality between sentences.

The following subsections describe how the proposed mining algorithm extends the classical association rule mining to acquire both intra- and inter-sentential language patterns.

### Intra-sentential language pattern mining

This section describes two methods for generating intra-sentential language patterns: extended association rule mining and sentence parsing.

### Method 1: extended association rule mining

For the mining of intra-sentential language patterns, rather than mining frequent item sets in the classical association rule mining problem, we attempt to mine frequent word sets (frequently co-occurred words) in the sets of cause and effect text spans. For this purpose, we adopted a modified version of the Apriori algorithm [24,31,32]. The basic concept behind the Apriori algorithm is the recursive identification of frequent word sets from which intra-sentential language patterns are then generated. For simplicity, only nouns and verbs are considered in language pattern generation. The detailed procedure is described as follows.

### Find frequent word sets within cause and effect text spans

A word set is frequent if it possesses a minimum level of support. The support of a word set is defined as the number of times the word set occurs in the set of cause (or effect) text spans. For instance, the support of a two-word set {*w*_*i*_,*w*_*j*_} denotes the number of times the word pair (*w*_*i*_,*w*_*j*_) occurs in the set of cause (or effect) text spans. The frequent *k*-word sets are discovered from (*k*-1)-word sets. First, the support of each word (i.e., the word frequency) was counted from the set of cause (or effect) text spans. The set of frequent one-word sets, denoted as *L*_1_, was then generated by choosing the words with a minimum support level. To calculate *L*_k_, the following two-step process is performed iteratively until no more frequent *k*-word sets are found.

· **Join step:** A set of candidate *k*-word sets, denoted as *C*_k_, is first generated by merging frequent word sets of *L*_k-1_, in which only the word sets with identical first (*k*-2) words can be merged.

**Figure 2:**
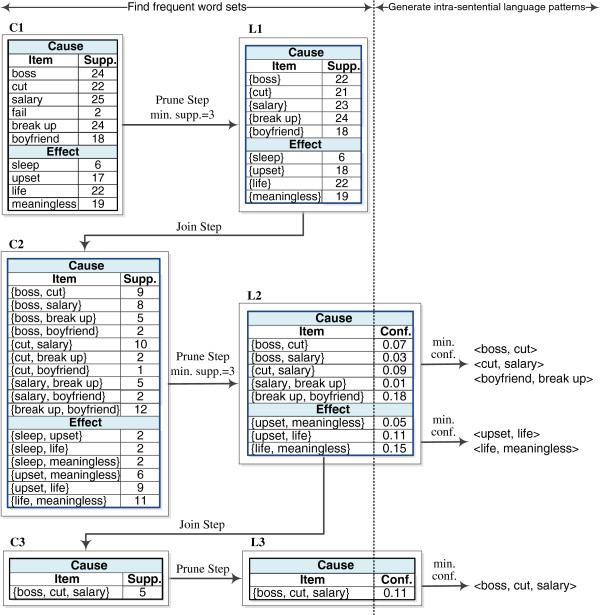
Generating intra-sentential language patterns for both cause and effect events.

· **Prune step:** The support of each candidate word set in *C*_k_ is then counted to determine which candidate word sets are frequent. Finally, the candidate word sets with a support count greater than or equal to the minimum support form *L*_k_. The candidate word sets with infrequent subsets were eliminated. Figure 2 shows an example of generating *L*_k_. The maximum value of *L*_k_ is determined when no more frequent *k*-word sets are found in the generation process.

### Generate intra-sentential language patterns from frequent word sets

Once the frequent word sets have been identified, the intra-sentential language patterns can be generated via a confidence measure. Let $lp_{i}=<w_{1},\ldots ,w_{k}>$ denotes an intra-sentential language pattern of *k* words. The confidence of $lp_{i}$ is defined as the mutual information of the *k* words [33-35], as shown below:

(1) $$\begin{array}{l} Conf(lp_{i})=MI(w_{1},\ldots w_{k})\\ =P(w_{1},\ldots w_{k})log\frac{P(w_{1},\ldots w_{k})}{\prod_{i=1}^{k}P(w_{i})} \end{array}$$

where $P(w_{1},\ldots w_{k})$ denotes the probability of the *k* words co-occurring in the set of cause (or effect) text spans, and $P(w_{i})$ denotes the probability of a single word occurring in the set of cause (or effect) text spans. Accordingly, for every frequent word set in *L*_k_, an intra-sentential language pattern is generated if the mutual information of the *k* words is greater than or equal to a minimum confidence. The resulting intra-sentential language patterns are those with a minimum confidence level. Figure 2 shows an example of generating intra-sentential language patterns from *L*_k_.

### Method 2: sentence parsing

**Figure 3:**
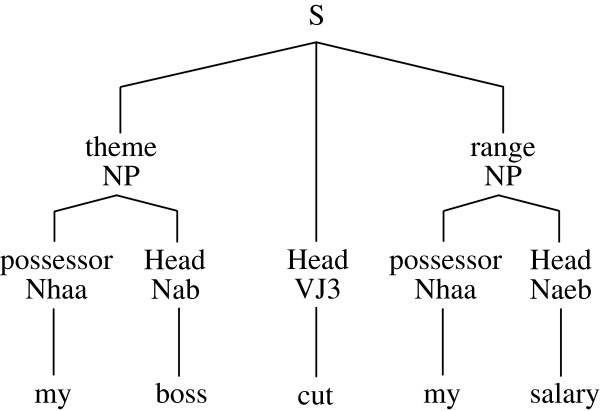
Example of a parse tree.

In addition to the extended association rule mining presented above, sentence parsing that considers sentence structure can also be used to discover word dependencies in sentences. Therefore, this study uses a parser developed by Academia Sinica, Taiwan [36] to generate intra-sentential language patterns by deriving word pairs with proper dependencies from the parse trees of both cause and effect text spans. Figure 3 shows the parse tree output for the sample sentence: *My boss cut my salary*.

The parser assigns a phrase label (e.g., NP, VP, PP, etc.) and a semantic label (e.g., Head, possessor, theme, etc.) to each constituent in the sentences. The dependencies of each word and its head are then considered as the intra-sentential language patterns. For example, in Figure 3, the intra-sentential language patterns for the sample sentences include (my, boss), (my, salary), (boss, cut), and (salary, cut).

### Inter-sentential language pattern mining

An *inter*-sentential language pattern is composed of at least one *intra*-sentential language pattern for cause events and one for effect events. Therefore, once the intra-sentential language patterns for cause and effect events are generated using each of the abovementioned methods, the next step is to generate inter-sentential language patterns by finding frequently co-occurring patterns between the cause and effect text spans. This can be accomplished by repeating the same procedure presented above for extended association rule mining to find frequent pattern sets which are then used to generate inter-sentential language patterns.

### Find frequent pattern sets between cause and effect text spans

**Figure 4:**
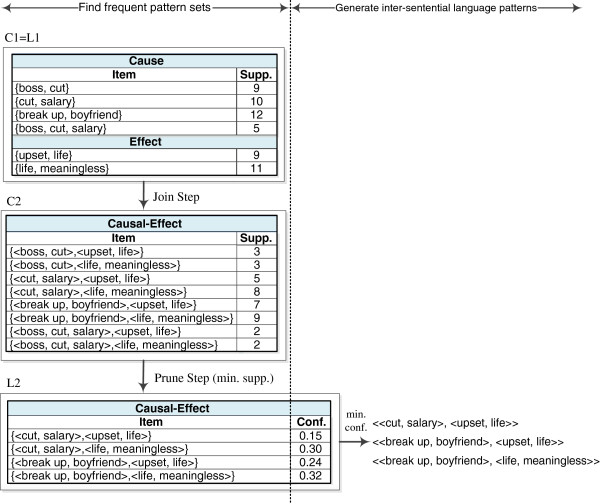
Generating inter-sentential language patterns for cause-effect relations.

The procedure for finding frequent pattern sets only differs from that of finding frequent word sets in terms of the definition of the support measure. In finding frequent word sets, the support of a word set is defined as the number of times the word set occurs in the set of cause (or effect) text spans. In this step, a pattern set is composed of at least one pattern from cause events and one from effect events. Therefore, the support of a pattern set is defined as the number of times the pattern set occurs between the sets of cause and effect text spans. For instance, suppose a two-pattern set {*lp*_*i*_,*lp*_*j*_} where *lp*_*i*_ and *lp*_*j*_ respectively denote an intra-sentential language pattern for the cause and effect events. The support of this two-pattern set is the number of times, *lp*_*i*_ and *lp*_*j*_ co-occur between the sets of cause and effect text spans. Therefore, in searching for frequent pattern sets, all combinations of the intra-sentential language patterns for the cause and effect events are considered as candidate pattern sets. The join and prune steps presented in the previous section can then be repeated to determine frequent pattern sets from all possible pattern combinations. Figure 4 shows an example.

### Generate inter-sentential language patterns from frequent pattern sets

Similar to the procedure for generating intra-sentential language patterns, this step requires a confidence measure to generate inter-sentential language patterns from frequent pattern sets. In generating intra-sentential language patterns, the confidence score is used to measure the mutual information of the words in a frequent word set. In this step, the confidence score is used to measure the mutual information of the patterns in a frequent pattern set. Let $islp_{i}=<lp_{1},\ldots ,lp_{k}>$ denotes an inter-sentential language pattern of *k* patterns. The confidence of *islp*_*i*_ is defined as the mutual information of the *k* words, as shown below:

(2) $$\begin{array}{l} Conf(islp_{i})=MI(lp_{1},\ldots ,lp_{k})\\ =P(lp_{1},\ldots ,lp_{k})log\frac{P(lp_{1},\ldots ,lp_{k})}{\prod_{i=1}^{k}P(lp_{i})} \end{array}$$

where $P(lp_{1},\ldots ,lp_{k})$ denotes the probability of the *k* patterns co-occurring between the sets of cause and effect text spans, and $P(lp_{i})$ denotes the probability of a pattern occurring in the set of cause (or effect) text spans. The resulting inter-sentential language patterns are those with a minimum confidence score. Figure 4 shows an example.

### Causality detection

This section describes the use of inter-sentential language patterns to detect causality between sentences, focusing on the detection of implicit cause-effect relations. Other studies have also demonstrated the use of surface text patterns for relation extraction [37,38]. Given a sentence pair (*s*_*i*_*s*_*j*_) without any discourse connective between *s*_*i*_ and *s*_*j*_, the goal is to classify the sentence pair into causality or non-causality, as shown below:

(3) $$\begin{array}{l} c^{*}=\underset{c_{k}}{argmax}P(c_{k}|s_{i},s_{j})\\ =\underset{c_{k}}{argmax}P(s_{i},s_{j}|c_{k})P(c_{k})\text{,} \end{array}$$

where c* is the prediction output, representing causality (*c*_*k*=1_) or non-causality (*c*_*k*=0_). Before prediction, the input sentence pair (*s*_*i*_*s*_*j*_) is first transformed into feature representation. This study uses both inter-sentential language patterns and previously proposed word pairs as features. As each sentence pair is transformed into pattern representation, it is represented by a single or multiple inter-sentential language patterns depending on the number of patterns the sentence pair matched in the set of discovered inter-sentential language patterns. Therefore, a sentence pair containing *n* inter-sentential language patterns can be formally represented as $\left(s_{i},s_{j})=ISLP_{s_{i},s_{j}}=\{islp_{1},\ldots ,islp_{n}\right\}$. In the word-pair representation, each sentence pair is represented by a set of word pairs, denoted as $\left(s_{i},s_{j})=WP_{s_{i},s_{j}}=\{(w_{i},w_{j})|w_{i}\in s_{i}\text{,}\;w_{j}\in s_{j}\right\}$. By using these two features, Eq. (3) can be re-written as

(4) $$c^{*}=\underset{c_{k}}{argmax}P(ISLP_{s_{i},s_{j}},WP_{s_{i},s_{j}}|c_{k})P(c_{k})\text{.}$$

where $ISLP_{s_{i},s_{j}}$ and $WP_{s_{i},s_{j}}$ represent the feature sets of inter-sentential language patterns and word pairs of the input sentence pair $\left(s_{i},s_{j}\right)$, respectively. Assume that $ISLP_{s_{i},s_{j}}$ and $WP_{s_{i},s_{j}}$ are independent. Eq. (4) can be re-written as

(5) $$c^{*}=\underset{c_{k}}{argmax}P(ISLP_{s_{i},s_{j}}|c_{k})P(WP_{s_{i},s_{j}}|c_{k})P(c_{k})\text{.}$$

Assuming again that the elements in both $ISLP_{s_{i},s_{j}}$ and $WP_{s_{i},s_{j}}$ are independent, then

(6) $$c^{*}=\underset{c_{k}}{argmax}\prod_{i=1}^{n}P(islp_{i}|c_{k})\prod_{w_{i}\in s_{i}\text{,}\;w_{j}\in s_{j}}P((w_{i},w_{j})|c_{k})P(c_{k})\text{.}$$

where $P(islp_{i}|c_{k})$ and $P((w_{i},w_{j})|c_{k})$ denote the respective probabilities of an inter-sentential language pattern and a word pair occurred in the causality or non-causality class, and $P(c_{k})$ denotes the probability of the causality or non-causality class. These probabilities can be estimated from the training data:

(7) $$P(islp_{i}|c_{k})=\frac{N(islp_{i},c_{k})}{N(c_{k})}\text{,}$$

(8) $$P((w_{i},w_{j})|c_{k})=\frac{N(w_{i},w_{j},c_{k})}{N(c_{k})}\text{,}$$

(9) $$P(c_{k})=\frac{N(c_{k})}{N}\text{,}$$

where $N(islp_{i},c_{k})$ and $N(w_{i},w_{j},c_{k})$ denote the respective frequency counts of an inter-sentential language pattern and a word pair occurring in the causality or non-causality class, $N(c_{k})$ denotes the number of causality or non-causality sentences in the training data, and *N* denotes the total number of sentences in the training data.

## Results and Discussion

This section presents the experimental results for causality detection. We first explain the experimental setup, including experiment data, features used for causality detection, and evaluation metrics. The selection of optimal parameter settings for inter-sentential language pattern mining is then described, followed by the evaluation results of causality detection with different features.

### Experimental setup

· **Data:** A total of 9716 sentence pairs were collected from PsychPark [29,30], from which 8035, 481, and 1200 sentence pairs were randomly selected as the training set, development set, and test set, respectively. For each data set, a set of discourse connectives collected based on the results of previous studies [16,17], were used to select causality sentence pairs. The statistics of the data sets are presented in Table 1. The training set was used to generate the inter-sentential language patterns and word pairs. The validation set was used to select the optimal value of the parameters used in inter-sentential language pattern mining. The test set was used to evaluate the performance of causality detection.

· **Features used for causality detection:** This experiment used word pairs (WP) and inter-sentential language patterns (ISLP) as features to detect causality between sentences. For ISLP, we used ISLP_ARM_ and ISLP_parsing_ to denote the sets of inter-sentential language patterns generated from the intra-sentential language patterns respectively discovered using the extended association rule mining and sentence parsing. Thus, the causality detection method was implemented using three feature sets: WP, WP + ISLP_ARM_ and WP + ISLP_parsing_, where WP was used to construct a baseline for causality detection, while WP + ISLP_ARM_ and WP + ISLP_parsing_ were used to determine whether or not the newly proposed inter-sentential language patterns could further improve detection performance, and determine which method (i.e., extended association rule mining or sentence parsing) could generate intra-sentential language patterns more useful for subsequent inter-sentential language pattern mining for causality detection.

· **Evaluation metrics:** The metrics used for performance evaluation included *recall*, *precision*, and *F-measure*, respectively, defined as follows:

**Table 1:** Statistics of experimental data

**Data sets**	**Training set**	**Validation set**	**Test set**
Number of causality sentence pairs	2,835	236	472
Number of non-causality sentence pairs	5,200	245	728
Total	8,035	481	1,200

(10) $$Recall=\frac{\text{number of causality sentence pairs correctly identified by the method}}{\text{number of causality sentence pairs in the test set}}\text{.}$$

(11) $$Precision=\frac{\text{number of causality sentence pairs correctly identified by the method}}{\text{number of causality sentence pairs identified by the method}}\text{.}$$

(12) $$F-measure=\frac{2\times recall\times precision}{recall+precision}\text{.}$$

### Evaluation of inter-sentential language pattern mining

**Figure 5:**
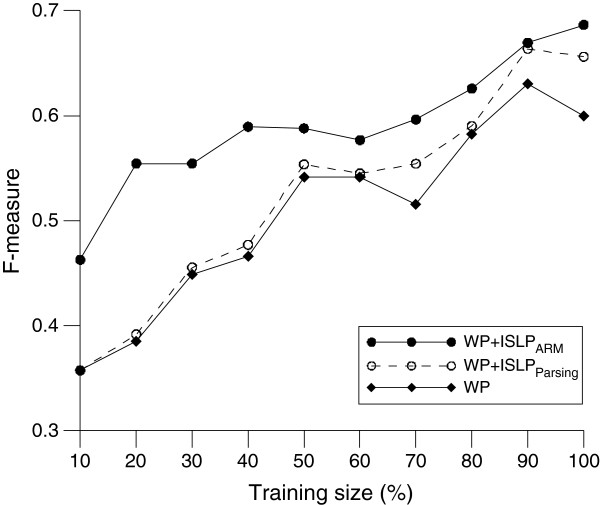
Performance against different proportions of training data.

In inter-sentential language pattern mining, two parameters may affect the quantity and quality of the discovered patterns: the size of training data and threshold value of confidence (Eq. (2)). The size of the training data set was used to control the number of documents used for pattern generation. The threshold value of confidence was used to control the number of patterns generated from training data. The optimal values of both parameters were determined by maximizing the performance of causality detection on the development set. Figure 5 shows the F-measure of causality detection for different proportions of training data. The results show that increasing the size of the training data set increased the performance of WP, WP + ISLP_ARM_, and WP + ISLP_parsing_, mainly because more useful features can be discovered from a larger training set.

**Figure 6:**
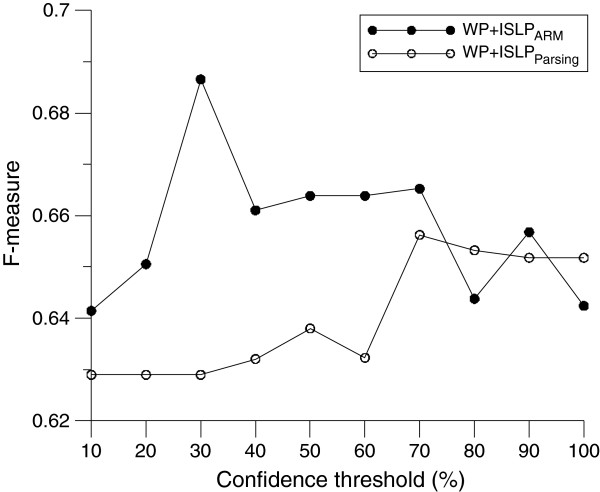
Performance against different threshold values of confidence.

For the confidence threshold, a higher value represents a more confident pattern. In the pattern generation process, all discovered patterns were sorted in descending order of their confidence values. A threshold percentage was then applied to select the top N percent of patterns for causality detection. Figure 6 shows the F-measure of causality detection for different percentages of selected patterns. The results show that for WP + ISLP_ARM_ performance increased as the threshold value increased to 0.3, indicating that the top 30 % of patterns were useful for detecting causality due to their higher level of confidence. When the threshold value exceeded 0.3, the performance decreased because the lower ranks contained more noisy patterns that tended to increase ambiguity in causality detection. For WP + ISLP_parsing_, the optimal threshold value was 0.7.

### Results of causality detection

This section presents the comparative results of using different feature sets for causality detection. The results presented in Table 2 were obtained from the test set with 10-fold cross validation, using the optimal parameter settings selected in the previous section. A paired, two-tailed *t*-test was used to determine whether the performance difference was statistically significant.

**Table 2:** Comparative results of causality detection with different feature sets

	**Recall**	**Precision**	**F-measure**
WP	58.60 %	63.38 %	60.83 %
WP + ISLP_Parsing_	61.11 %^**+**^	64.51 %	62.71 %^**+**^
WP + ISLP_ARM_	60.15 %^*^	66.54 %^*^	63.14 %^*^

^+^ ISLP_Parsing_ vs WP significantly different (p < 0.05).

^*^ ISLP_ARM_ vs WP significantly different (p < 0.05).

The row labeled WP indicates that it used word pairs alone as features, providing a baseline result for causality detection. Once inter-sentential language patterns were used, both WP + ISLP_Parsing_ and WP + ISLP_ARM_ improved the recall, precision, and F-measure over WP, indicating that the proposed inter-sentential language patterns are significant features for causality detection. As listed in Table 3, the inter-sentential language patterns are more semantically complete and can provide more precise information because they can capture the associations of multiple words within and between sentences. Conversely, word pairs such as (friend, energy) and (investment, life), which consider only individual word relationships, are usually semantically incomplete and ambiguous, thus yielding lower performance. Both WP + ISLP_Parsing_ and WP + ISLP_ARM_ achieved a similar F-measure, indicating that both extended association rule mining or sentence parsing can generate intra-sentential language patterns that are useful for subsequent inter-sentential language pattern mining for causality detection.

**Table 3:** Examples of inter-sentential language patterns

**No.**	**Inter-sentential language patterns**	
	**Cause**	**Effect**
1	<<friend, argue>,	<lose, energy>>
2	<<investment, setback>,	<life, trouble>>
3	<<performance, decrease>,	<change, job>>
4	<<parents, divorce>,	<family, break>>
5	<child, sick>,	<feeling, upset>>

## Conclusions

This study proposes the use of inter-sentential language patterns to detect cause-effect relations in online psychiatric texts. We also present a text mining framework to mine inter-sentential language patterns by associating frequently co-occurring language patterns across the sentence boundary. Experimental results show that using the proposed inter-sentential language patterns improved the performance above the use of word pairs alone, mainly because the inter-sentential language patterns are semantically more complete and can thus provide more precise information for causality detection. Future work will be devoted to investigating more useful cross-sentence features and information fusion methods to further improve system performance.

## Endnotes

^a^http://www.depressionforums.org/forums.

^b^http://www.psychpark.org.

^c^http://www.social-anxiety.org.uk.

^d^http://www.webmd.com.

^e^http://answers.yahoo.com.

## Competing interests

The author(s) declare that they have no competing interests.

## Authors’ contributions

JLW collected the corpus, designed the experiment, and contributed to writing the paper. LCY designed the study, interpreted experiment results, and contributed to writing the paper. PJC restructured the paper and contributed to writing the paper. All of authors read and approved the final manuscript.

## Pre-publication history

The pre-publication history for this paper can be accessed here:

http://www.biomedcentral.com/1472-6947/12/72/prepub

## References

[B1] Eysenbach G Medicine 2.0: Social Networking, Collaboration, Participation, Apomediation, and Openness J Med Internet Res 2008 10 3 e22 10.2196/jmir.1030 18725354 PMC2626430

[B2] Huang CM Chan E Hyder AA Web 2.0 and Internet Social Networking: A New tool for Disaster Management? - Lessons from Taiwan BMC Med Inform Decis Mak 2010 10 57 10.1186/1472-6947-10-57 20925944 PMC2958996

[B3] Yardley L Morrison LG Andreou P Joseph J Little P Understanding reactions to an internet-delivered health-care intervention: accommodating user preferences for information provision BMC Med Inform Decis Mak 2010 10 52 10.1186/1472-6947-10-52 20849599 PMC2946266

[B4] Kleinberg S Hripcsak G A review of causal inference for biomedical informatics J Biomed Inform 2011 44 6 1102 1112 10.1016/j.jbi.2011.07.001 21782035 PMC3219814

[B5] Girju R Moldovan D Mining answers for causation Proceedings of the AAAI Spring Symposium 2002 AAAI Press, Stanford, CA, USA 15 25

[B6] Niu Y Hirst G Analysis of semantic classes in medical text for question answering Proceedings of the ACL 2004 Workshop on Question Answering in Restricted Domains 2004 Association for Computational Linguistics, Barcelona, Spain

[B7] Demner-Fushman D Lin J Answering clinical questions with knowledge-based and statistical techniques Comput Linguist 2007 33 1 63 103 10.1162/coli.2007.33.1.63

[B8] Mulkar-Mehta R Hobbs JR Liu CC Zhou XJ Discovering causal and temporal relations in biomedical texts Proceedings of the AAAI Spring Symposium 2009 AAAI Press, Stanford, CA, USA 74 80

[B9] Boudin F Nie JY Bartlett JC Grad R Pluye P Dawes M Combining classifiers for robust PICO element detection BMC Med Inform Decis Mak 2010 10 29 10.1186/1472-6947-10-29 20470429 PMC2891622

[B10] Prasad R McRoy S Frid N Joshi A Yu H The biomedical discourse relation bank BMC Bioinformatics 2011 12 188 10.1186/1471-2105-12-188 21605399 PMC3130691

[B11] Radinsky K Davidovich S Markovitch S Learning causality from textual data Proceedings of the IJCAI Workshop on Learning by Reading and its Applications in Intelligent Question-Answering 2011 AAAI Press, Barcelona, Spain 363 367

[B12] Yu LC Wu CH Jang FL Psychiatric document retrieval using a discourse-aware model Artif Intell 2009 173 7–8 817 829

[B13] Faghihi U Fournier-viger P Nkambou R A computational model for causal learning in cognitive agents Knowl-based Syst 2012 30 48 56

[B14] Hobbs JR On the coherence and structure of discourse, Report No. CSLI-85-37. Center for the Study of Language and Information 1985 Stanford University Press, California

[B15] Power R Scott D Bouayad-Agha N Document structure Comput Linguist 2003 29 2 211 260 10.1162/089120103322145315

[B16] Wolf F Gibson E Representing discourse coherence: a corpus-based study Comput Linguist 2005 31 2 249 287 10.1162/0891201054223977

[B17] Wu CH Yu LC Jang FL Using semantic dependencies to mine depressive symptoms from consultation records IEEE Intell Syst 2005 20 6 50 58 10.1109/MIS.2005.115

[B18] Ramesh BP Yu H Identifying discourse connectives in biomedical text Proceedings of the AMIA 2010 Symposium: 22–26 Oct 2010 2010 American Medical Informatics Association, Washington, DC 657 661 PMC3041460 21347060

[B19] Inui T Inui K Matsumoto Y Acquiring causal knowledge from text using the connective markers J Inf Process Soc Jpn 2004 45 3 919 993

[B20] Rink B Bejan CA Harabagiu S Learning textual graph patterns to detect causal event relations Proceedings of the 23rd International Florida Artificial Intelligence Research Society Conference 2010 AAAI Press, Daytona Beach, Florida, USA 265 270

[B21] Mulkar-Mehta R Welty C Hobbs JR Hovy EH Using Part-Of relations for discovering causality Proceedings of the 24th International Florida Artificial Intelligence Research Society Conference 2011 AAAI Press, Palm Beach, Florida, USA 57 62

[B22] Marcu D Echihabi A An unsupervised approach to recognizing discourse relations Proceedings of the 40th Annual Meeting on Association for Computational Linguistic, ACL’02 2002 Association for Computational Linguistics, Philadelphia, PA, USA 368 375

[B23] Chang DS Choi KS Incremental discourse connective learning and bootstrapping method for causality extraction using discourse connective and word pair probabilities Inf Process Manage 2006 42 3 662 678 10.1016/j.ipm.2005.04.004

[B24] Agrawal R Srikant R Fast algorithms for mining association rules Proceedings of the 20th International Conference Very Large Data Bases 1994 Morgan Kaufmann Publishers Inc., Hong Kong, China 487 499

[B25] Tai YM Chiu HW Comorbidity study of ADHD: applying association rule mining (ARM) to National Health Insurance Database of Taiwan Int J Med Inform 2009 78 12 e75 e83 10.1016/j.ijmedinf.2009.09.005 19853501

[B26] Hu H Mining patterns in disease classification forests J Biomed Inform 2010 43 5 820 827 10.1016/j.jbi.2010.06.004 20601123

[B27] Herawan T Mat Deris M A soft set approach for association rules mining Knowl-based Syst 2011 24 1 186 195 10.1016/j.knosys.2010.08.005

[B28] Liu H Lin F He J Cai Y New approach for the sequential pattern mining of high-dimensional sequence databases Decis Support Syst 2010 50 1 270 280 10.1016/j.dss.2010.08.029

[B29] Bai YM Lin CC Chen JY Liu WC Virtual psychiatric clinics Am J Psychiat 2001 158 7 1160 1161 10.1176/appi.ajp.158.7.1160 11431247

[B30] Lin CC Bai YM Chen JY Reliability of information provided by patients of a virtual psychiatric clinic Psychiat Serv 2003 54 8 1167 1168 10.1176/appi.ps.54.8.1167 12883154

[B31] Chien JT Association pattern language modeling IEEE Trans Audio Speech Lang Process 2006 14 5 1719 1728

[B32] Wu CH Chuang ZJ Lin YC Emotion recognition from text using semantic labels and separable mixture models ACM Trans. Asian Lang Inf Process 2006 5 2 165 182 10.1145/1165255.1165259

[B33] Church K Hanks P Word association norms, mutual information and lexicography Comput Linguist 1991 16 1 22 29

[B34] Manning C Schütze H Foundations of Statistical Natural Language Processing 1999 MIT Press, Cambridge, MA

[B35] Yu LC Chien WN Chen ST A baseline system for Chinese near-synonym choice Proceedings of the 5th International Joint Conference on Natural Language Processing, IJCNLP’11 2011 Asian Federation of Natural Language Processing;, Chiang Mai, Thailand 1366 1370

[B36] Hsieh YM Yang DC Chen KJ Linguistically-motivated grammar extraction, generalization and adaptation Proceedings of the Second International Joint Conference on Natural Language Processing, IJCNLP’05 2005 Springer, Jeju Island, Korea 177 187

[B37] Ravichandran D Hovy EH Learning surface text patterns for a question answering system Proceedings of the 40th Annual Meeting on Association for Computational Linguistic, ACL’02 2002 Association for Computational Linguistics, Philadelphia, PA, USA 41 47

[B38] Bhagat R Ravichandran D Large scale acquisition of paraphrases for learning surface patterns Proceedings of the 46th Annual Meeting on Association for Computational Linguistic: Human Language Technologies, ACL’08: HLT 2008 Association for Computational Linguistics, Columbus, OH, USA 674 682

